# Origins of the method of standard additions and of the use of an internal standard in quantitative instrumental chemical analyses

**DOI:** 10.1007/s00216-019-01754-w

**Published:** 2019-04-02

**Authors:** D. Thorburn Burns, Michael J. Walker

**Affiliations:** 1 Institute for Global Food Security, The Queen’s University of Belfast, Belfast, BT9 5AG UK; 2 Laboratory of the Government Chemist, Teddington, Middlesex, TW 11 0LY UK

**Keywords:** History, Internal standards, Method of standard additions

## Abstract

The origin of the method of standard additions (SAM) and of the use of internal standard (IS) in instrumental chemical analysis and their spread into other areas has been reviewed. Recorded applications of IS range from flame spectroscopy in 1877 through multiple techniques to current use in NMR and standard additions with isotopically labelled internal standards in hyphenated techniques. For SAM, applications stemmed from polarography in 1937 and spread to most instrumental methods. Some misconceptions on priorities are corrected.

## Introduction

The method of standard additions (SAM) and internal standard (IS) are regularly used and regarded as self-evident concepts to modern analytical chemists, thus apparently requiring no history. However, we will show both stem from the original work of several chemists whose priority should be properly acknowledged. The two methods are related by the addition of analytes to the sample but they are quite different; SAM uses known additions of the same analyte whilst IS uses a known amount of an easily measured analyte that is absent, or essentially absent, from the sample. The origin of both procedures is missing from most texts on instrumental methods of analysis [e.g. 1–6]. The histories of both procedures have been reviewed [7, 8], both are of interest in that they each have suffered from misrepresentations as to their originators and in their initial applications in various technique areas.

## Method of standard additions

### Polarography 1937 onwards

Burriel-Martí and Ramírez-Muños (1957) state [9] “This is a very simple method of obtaining experimental values, which has been known and widely used for a long time in all instrumental analyses (colorimetry, spectrography, polarography etc.), as it allows working with some accuracy without corrections and without adding reference elements (internal standards)”.

**Fig. 1:**
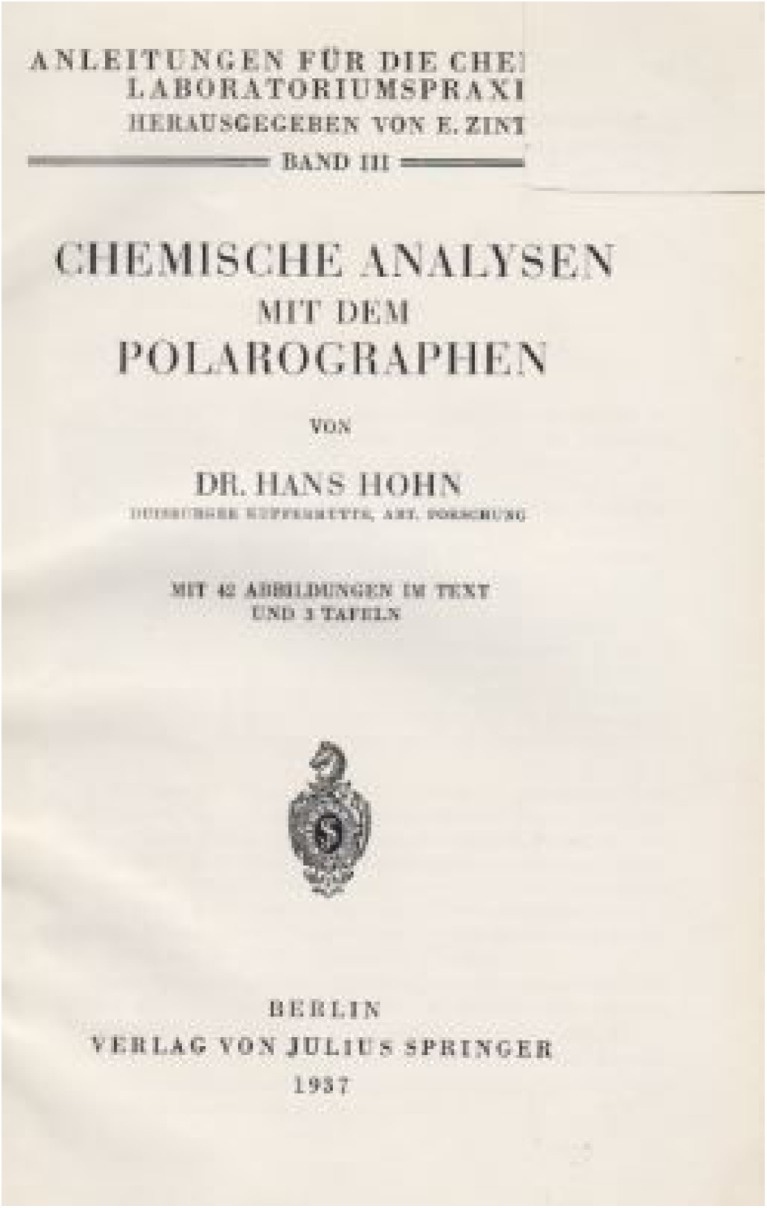
Title page, H. Hohn “Chemische Analysen mit dem Polarographem” [10]

However, Kelly et al. [7] have most usefully noted that the method was first used by Hans Hohn and described in 1937 in his book, *Chemische Analysen mit dem Polarographen* [10] (see Fig. 1) as *Die Methode des Eichzusatzes*. Kelly et al. show the original polarograms for the determination of zinc by addition in the presence of Cu, Tl and Mn, and the method of calculation allowing for the effect of dilution following the addition of the Zn standard solution. In this section of text, Hone makes reference to one of Heyrovský’s accounts of polarography [11] but as this does not mention the *eichzusatzes* method, the priority remains with Hohn. Hohn then described in detail the application of *eichzusätz* to the determination of trace elements (Cu, Pb, Zn and Fe) in an essentially pure sample of aluminium [10 a]. Hohn’s book was most favourably reviewed in the *Analyst* by Glasstone in 1938, who concluded “Every analyst who is contemplating the use of the polarographic method should certainly have a copy of this book; it will save both time and anxiety” [12]. The Hohn method was used, in the same year it was first described, by Varasova for the determination of both Cu and Ni in Cu-Ni ores [13].

Kelly et al. state that the first use of the term “standard addition” in English was by Lingane and Kerlinger in 1941 [14]. The early polarographers only used a single addition until Kemula in 1966 introduced multiple additions in what he called the “sluice addition method” [15, 16].

### Atomic spectrometry 1950 onwards

The first recent use of the method of standard additions outside the area of polarography was in atomic emission spectrometry as described in Harvey’s book, *Spectrochemical Procedures* [17], who referred to the method as an extrapolation method. The calibration graphs were presented by Harvey, not as used today, but with the axes reversed, using the abscissa (*x* axis) for instrument response, and the ordinate (*y* axis) for the addition amounts. Neither Lingane and Kerlinger, Kemula nor Harvey made reference to the work of Hohn.

Kelly et al. discussed the priority for the use of SAM beyond the field of polarography noting its use in emission spectrography by Harvey, reported in 1950 [17]. However, their analysis of priority between Campbell and Carl’s use in *X-ray fluorescence* (1954) [18] and Chow and Thompson’s use in *flame photometry* (1955) [19] is rendered pointless in the light of Chow and Thompson’s reference to Smales [20] (their reference [10]). The incorrect priority given to Chow and Thompson’s work was first stated in 2008 [21].

Smales in 1951 used the method of standard additions in flame photometry, interestingly plotting the data in the same manner as Harvey [17] but with no reference to his reasons, or reference to Harvey or to Hohn [10]. The paper by Smales was also missed by Mavrodineanu and Boiteux [22], who noted Harvey, but said erroneously that SAM was first applied in flame spectroscopy in 1957 by Fukushima et al. [23].

The *standard addition-logarithmic extrapolation* is available when the emission intensity is not linear with concentration in arc and spark [24] and in flame excitation [25]. In 1980, Bader [26] discussed the possible errors in the use of SAM and its application to *potentiometry* and to *isotope dilution analysis* but again gave no reference to Hohn. Ellison and Thompson have discussed the myth and reality of SAM [27] and Anderson has recently revisited the SAM to review its performance parameters [28].

### ^1^H NMR 2012 onwards

In a review of *Quantitative*^*1*^*H NMR spectroscopy*, the use of both SAM and IS was outlined [29].

### UV-visible spectrometry 1960 onwards

Other than the tangential reference in 1957 to “colorimetry” [9] and despite extensive searches via *SciFind*er and journals (manually), it has not so far been possible to locate the first clear use of SAM in UV-visible spectrometry. The late adoption in UV-visible spectrophotometry is probably due to the intensive interest by inorganic analysts in the 1950s in new and selective colorimetric reagents and in the masking of interfering ions. Organic analysts used separations or corrected for irrelevant absorptions in the ultraviolet region using the Morton and Stubbs procedure [30] and its subsequent developments [31]. The earliest reference to hand, 1960, concerns the separation of microgram amounts of cobalt from nickel followed by determination with nitroso-R salt at 529 nm, no details were given, only the statement, “to estimate the efficiency of the method described, the method of standard addition of cobalt was followed” [32]. In 1961, a single standard addition procedure was published for the determination of sodium ethyl-mercurithiosalicylate [33].

## Use of internal standards

### Flame spectroscopy 1877 onwards

**Fig. 2:**
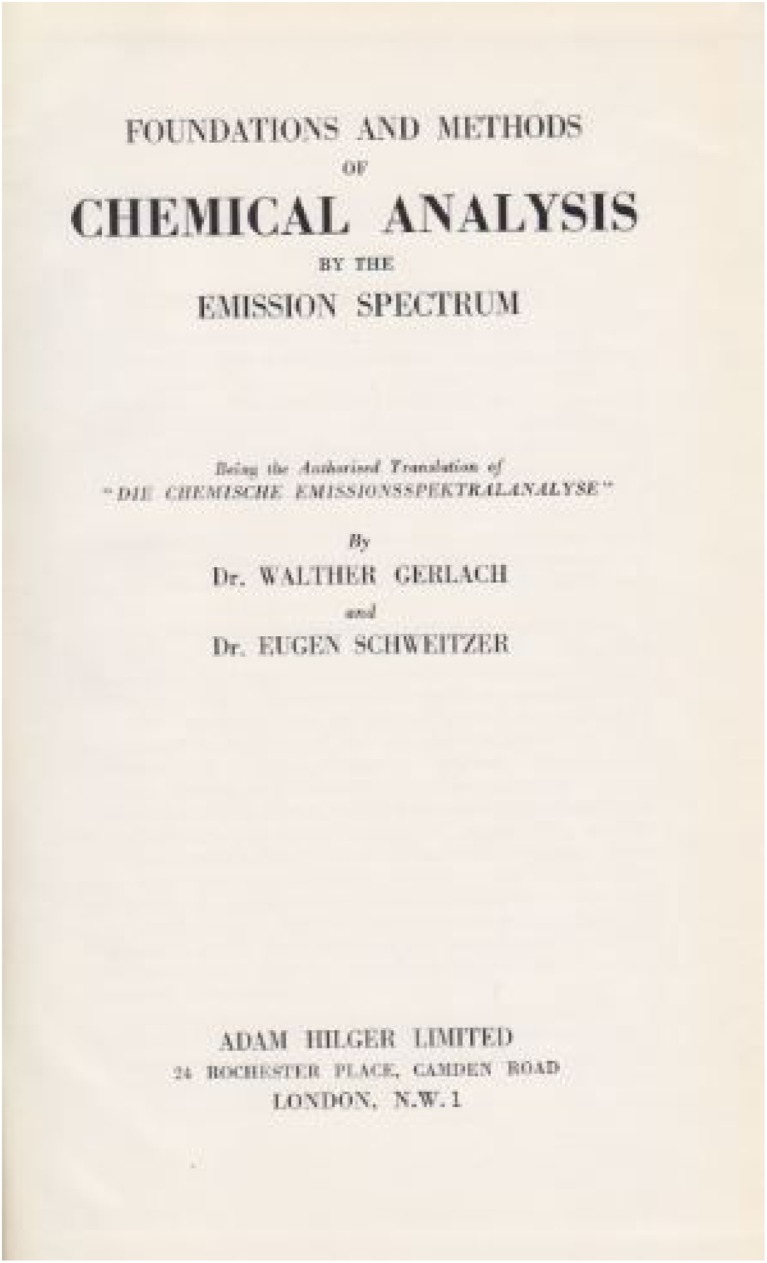
Title page of W. Gerlach and E. Schweitzer [36]

The first recorded use of IS was in *flame spectroscopy* in 1877 [34], by Gouy, who used it to verify the constancy of excitation in his flame. The procedure reappeared when commercial recording flame photometers became available in the 1940s [35]. IS was introduced in arc and spark *emission spectrometry* to deal with problems including excitation source instability and variable photographic film sensitivity [8] by the extensive studies by Gerlach using homologous line pairs and fixation pairs [36] (see Fig. 2) and is described in Harvey’s book [17]. Feldman in his report of experimental study of IS and the factors that influence signals in *atomic emission* and *absorption spectrometry* such as fuel-air flow, surface tension and aspiration rate [37] states that the first use of IS in AAS was in 1965 by Butler and Strasheim [38].

### Chromatography 1954 onwards

The IS method is widely used in *gas* and in *liquid chromatography* for several reasons including avoidance of the need for measuring very accurately sample injection volumes and to compensate for sample losses between injection and detection and variable detector response. In Strouts et al. [39], the use of IS in GLC they cite was that by Ray in 1954 [40]. An early example of the use of IS in HPLC was in the determination of benzenepolycarboxylic acids [41].

### ^1^H NMR 2012 onwards

The first quantitative applications of ^1^H NMR were described in 1963 [42, 43]. The addition of the internal standard directly to the sample can be avoided in quantitative *NMR* by using a co-axial stem insert to the NMR tube whereby the standard is surrounded by the sample and thus both measured under the same conditions [29]. The selection of appropriate standards has for quantification by ^1^H NMR been discussed by Rundlöf et al. [44]. The progress of NMR spectroscopy in food analysis is the subject of an ongoing RSC series “Magnetic resonance in food science” and has been reviewed for food analysis in particular [45]. The recent availability of low-cost, bench-top ^1^H NMR has produced useful data on foodstuffs, for example on the origin [46] and on the adulteration of honey [47].

### Spectrometry and spectroscopy1947 onwards

The problems with and the choice of appropriate IS in quantitative *Raman spectrometry* have been outlined [48]. An early example, if not the first, is that of antimony trioxide in the determination of the amounts of orthorhombic and tetragonal phases in samples of lead monoxide [49]. The use of an IS in quantitative *infrared spectroscopy* to avoid needing to determine sample thickness when using Nujol mulls dates back to 1947 [50] and 10 years later, for potassium bromide discs [51].

### Routine applications

**Fig. 3:**
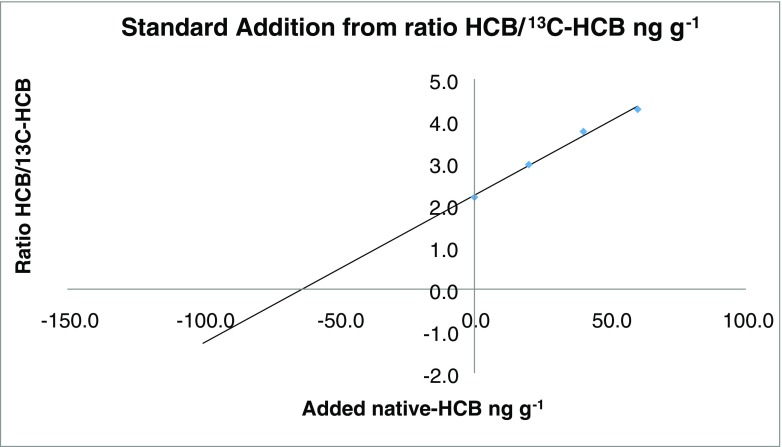
Example of combined SAM/IS in GC-MS of hexachlorobenzene in tea (one of multiple such experiments from which the final result was derived)

SAM combined with isotopically labelled IS is routinely applied in many laboratories including referee casework in the Laboratory of the Government Chemist where the utmost analytical rigour is required. The determination of the pesticide hexachlorobenzene in tea is illustrated as an example (Fig. 3) where the result is easily obtained from the linear regression function in Microsoft Excel. Innovation in SAM persists as shown by a recent study on a generic standard additions method in immunoassays to overcome complex biological matrix interference and accommodate non-linearity [52].

## Conclusions

Both SAM and IS continue to be of great utility to analytical chemists. However, for the sake of historical accuracy and due acknowledgement of priority of invention, students should be taught the origins of the method of standard additions and that of the use of internal standards in chemical analysis.

The first use of a standard addition method in instrumental chemical analysis was that in polarography by Hohn in 1937. There have been some misunderstandings in the literature about its first use in other technique areas.

The first use of an internal standard was that by Gouy in 1887 in flame emission spectroscopy. Following the extensive studies by Gerlach of the sensitive multi-element emission spectrometric analysis, in the late 1920s, and subsequent appreciation of the concept, it has spread into numerous other technique areas.

We await with interest any published applications of SAM and/or IS in analytical molecular biology although here the specificity of PCR may render its use unnecessary in routine applications.
